# Effects of Anthropometric Growth and Basketball Experience on Physical Performance in Pre-Adolescent Male Players

**DOI:** 10.3390/ijerph17072196

**Published:** 2020-03-25

**Authors:** Natascia Rinaldo, Stefania Toselli, Emanuela Gualdi-Russo, Nicoletta Zedda, Luciana Zaccagni

**Affiliations:** 1 Department of Biomedical and Specialty Surgical Sciences, Faculty of Medicine, Pharmacy and Prevention, University of Ferrara, 44121 Ferrara, Italy; natascia.rinaldo@unife.it (N.R.); zddnlt@unife.it (N.Z.); luciana.zaccagni@unife.it (L.Z.); 2 Department of Biomedical and Neuromotor Sciences, Faculty of Exercise and Sport Sciences, University of Bologna, 40126 Bologna, Italy; 3 Biomedical Sport Studies Center, University of Ferrara, 44121 Ferrara, Italy

**Keywords:** sports practice, growth, pre-adolescents, team sports, anthropometry, motor tests

## Abstract

During pre-adolescence, several variables connected to growth and weight status could influence physical performance and technical skills. This study aims to assess the influence of growth-related anthropometric changes, weight status, and basketball experience on physical performance in pre-adolescent basketball players. The sample consisted of 50 male basketball players (12.2 ± 0.4 years-old) included in the U-13 category. Anthropometric characteristics were collected using standard procedures. Physical performance was evaluated by 20 m Dash, *T*-test, squat jump, countermovement jump, and medicine ball throw. In order to assess growth-related changes, we conducted two surveys within a 10-week time span. Student’s *t*-test was used to compare the data collected in the two surveys and multiple regression analyses were performed to assess the effects of growth, weight status, and basketball experience on performance. After 10 weeks there was a significant increase in FFM (Fat-Free Mass) and a significant decrease in fat parameters. Moreover, between the two surveys, all motor tests significantly improved. The results of linear regression models showed that changes in %F (percentage of body fat) were significantly associated with speed and agility, while handgrip strength and weight status were associated with upper limb explosive strength. Basketball experience was a significant predictor of all three motor tests. In conclusion, body composition changes, years of experience, and weight status influenced physical performance and players’ motor skills ability, especially speed, agility, and upper limb explosive strength.

## 1. Introduction

Sports performance depends on a combination of physical, functional, and behavioral characteristics and sport-specific skills [1,2,3,4,5]. Even in the case of team sports, performance depends on different qualities, including body size, physical performance, and motor skills specific to each sport [6]. Basketball is one of the most popular team sports in the world [7] and elite players need high levels of strength, dexterity and speed [8,9,10,11], all of which require specific physical and anthropometric characteristics. In basketball, the importance of body size and body proportions, particularly concerning stature, arm span, and leg length, is well documented [6,8,9,10,12]. The physical performance markers can be important indicators of competitive success in young players [12] and anthropometric traits are fundamental for identifying and selecting talent. Moreover, there are other variables such as growth changes, basketball experience, training, and weight status that can affect the physical performance of young players. 

During the growth process, the relationship between anthropometric variables and physical performance have a higher level of complexity [13] and numerous differences can occur in the morphological growth and physiological development of players. Therefore, in preadolescent players, differences in growth rate and development can be a confounding factor affecting performance and success [14]. It is well known that adolescent growth and biological maturation strongly influence physical performance [12,15,16]. Young players with early maturation often outperform their late maturing peers in muscle strength and endurance. While numerous studies exist on the effects of biological maturation on the physical and technical performance of adolescent basketball players [12,15,16,17,18,19], there is a paucity of scientific literature on the assessment of anthropometric changes and performance characteristics in pre-adolescent basketballers. 

Sports training, if prescribed and supervised by professionals, is an effective and safe way to improve muscle strength in children and adolescents [20]. In general, sports training is associated with several health benefits, such as increased bone mineral density, better body composition, and improved mental health and well-being [20,21]. Sports training involves the development of physical, technical, tactical, and psychological characteristics [10], although it is not known which of these characteristics is the most prominent in terms of performance during a match [13,20]. Besides training, years of experience play an important role in performance. In their study, Gonzalez et al. (2013) [22] found that performance improved with the practice time by analyzing NBA players. Guimarães et al. (2019) [8] reported that years of experience provide the primary contribution to the variance in the technical skills tests. However, other studies in addition to that of Olvera et al. [23] are needed to investigate the relationship between years of experience and performance, especially in young basketball players.

Previous studies have found an association between nutritional and weight status and motor abilities in children. Well-fed children showed better results in the physical tests compared to malnourished children (both obese and under-nourished) [24]. 

If we consider only pre-adolescent players, how much do growth, training, and weight status influence their physical performance? We hypothesized that size increases, training, and lean weight during the growth process would pair with increasing performance.

This study aims, therefore, to investigate how physical performance assessed through motor tests is affected by sports experience and age-related morphometric changes during the first few months of training in a sample of 12-year-old basketball players. Furthermore, we also estimated which predictors (independent variables) were most strongly associated with performance outcomes (dependent variables) in multiple regression models. This analysis can provide useful indications for fitness assessment of young basketball players. Coaches and sports scientists could benefit from the findings of this study as reference data for further research on preadolescent basketball performance analysis models in relation to the anthropometric growth process.

## 2. Materials and Methods

### 2.1. Sample

We carried out this study on a sample of 50 pre-adolescent boys (age: 12.2 ± 0.4 years) from the under-13 basketball category. All the boys who attended two basketball schools (VIS Ferrara and Scuola Basket Ferrara) in Ferrara (northern Italy) volunteered to participate in the study. Written informed consent was provided by the parents before the study began. 

Players were excluded if they had any health problems that could have interfered with anthropometric measurements or the execution of motor tests. We also decided to exclude players who were not present at both surveys or who had an illness or injury resulting in loss of training during the period between the two surveys.

In addition to basketball tournament matches, the players trained for four and a half hours a week (three workouts of 1.5 h each). All of them also practiced another two hours of physical activity at school. On average, they had 5.7 ± 2.2 years of basketball experience.

The first survey was carried out at the beginning of the 2018–2019 basketball season, in September. The follow-up survey was conducted 10 weeks later, from the end of November to early December. Only boys who participated in both surveys without any other reasons for exclusion were included in the research. Consequently, data was collected for 50 boys out of the initial 53 (final participation rate: 94.3%). 

The study was approved by the Bioethics Committee of the University of Bologna (Approval n. 25027, dated 13 March 2017).

### 2.2. Procedures

All anthropometric measurements and motor tests were conducted in an indoor basketball court for the purpose of maintaining a consistent surface. The same procedures were repeated 10 weeks later at the same time of day and under the same experimental conditions. We chose a period of 10 weeks because this corresponds approximately to the interval between the resumption of sports activities of basketball players under-13 after the summer break and the suspension of such activities for the winter holidays.

#### 2.2.1. Anthropometric Measurements

We collected five somatometric characteristics (stature, sitting height, weight, triceps skinfold thickness, mid-upper arm circumference) and one physiometric characteristic (grip strength of right and left hands) by standardized procedures [25,26,27]. We calculated the length of the lower limb by the difference between stature and sitting height, in addition to some anthropometric indices listed below.

Stature was measured to the nearest 0.1 cm with a Raven anthropometer (Raven Equipment Ltd., UK). The participants (barefoot) were measured in a straightened upright position with the head oriented on the Frankfurt plane [28].

Sitting height was measured to the nearest 0.1 cm with a Raven anthropometer (Raven Equipment Ltd., UK) after sitting the boy on a box of known height and using the stretch stature method with the head oriented in the Frankfurt plane: sitting height is calculated by subtracting the height of the box from the stature value of participants.

Weight was measured to the nearest 0.1 kg using a Seca weighing scale (Seca Deutschland Medical Measuring Systems and Scale, Hamburg, Germany) on participants dressed in light clothing.

Triceps skinfold thickness (TST) was measured to the nearest 0.5 mm using a Lange skinfold caliper (Beta Technology Inc., Houston, TX, USA) by the same trained operator (N.R.) according to the guidelines of the International Biological Program [25]. The mean of two consecutive measurements was used in our analysis.

Mid-upper arm circumference (MUAC) was measured to the nearest 0.1 cm with an anelastic tape (GPM measuring tape, DKSH, Swiss) on the same level (midway between the tip of the acromion and olecranon processes) of TST on the participant in a relaxed position with arms along their sides.

Both bilateral somatometric measures (TST and MUAC) have been taken on the left side.

Body mass index (BMI) was computed as weight (kg)/stature squared (m^2^) and participants were divided into four categories (underweight, normal weight, overweight, and obese) according to Cole cutoff values by sex and age [29,30].

The body fat percentage (%F) was estimated according to Frerichs et al. [31] as follows:

%F = 51.73 + [0.28 × Weight (kg)] + [− 0.35 × Stature (cm)] + [0.78 × TST (mm)].(1)

The fat mass (FM), in kg, was computed as: %F × Weight/100.

Once the FM (kg) was obtained, the fat-free mass (FFM) in kg was derived by difference (Weight – FM).

To complete the body composition assessment, Frisancho’s formulas [32] from MUAC and TST have also been applied:

Upper arm muscle area (UMA) (cm^2^) = {MUAC-(TST × π)}^2^/(4 × π). (2)

Upper arm fat area (UFA) (cm^2^) = {(MUAC)^2^/(4 × π)}−UMA. (3)

Arm fat index (AFI) (%) = UFA/{(MUAC)^2^/(4 × π)} × 100. (4)

Handgrip (HG) strength was measured in kg using a hand grip dynamometer with a precision of 0.5 kg (Takei Scientific Instruments Co., Ltd., Niigata, Japan). The test was performed with the individual in a standing position with his arm at his side. The higher value of two trials, measured on each hand after a rest period (1 min), was used in the statistical analysis.

#### 2.2.2. Motor Tests

We tested speed (20 m dash) and agility (*T*-test) and lower and upper limb explosive strength (lower limb: squat jump, countermovement jump; upper limb: medicine ball throw). The assessment was carried out one week after players were familiarized with the testing procedures. Before testing, subjects were allowed to perform individual warm-ups for 10–15 min.

The run times for 20 m, at the maximum effort of participants, were measured (20 m dash) using a digital chronometer to the nearest 0.01 s. The start and finish lines were clearly indicated by cones. Each participant performed two speed run tests with a rest period of 3 min between them [13]. The best performance between two repeated sprint tests was recorded.

The *T*-test was performed according to Pauole et al. [33] and Sassi et al. [34] and included forward, sideways, and backward running. The time was measured with a digital chronometer to the nearest 0.01 s. The faster time of the two tests was used for the analysis.

A power endurance jump test was assessed by squat jump (SJ) and countermovement jump (CMJ) on a contact mat [35]. The Ergojump Bosco-System (MA.GI.CA., Rome, Italy) on the basis of flight times from take-off to landing immediately after the jump assesses directly the height of the jump (cm) by a microprocessor connected to the contact mat. The tests were carried out in accordance with Bosco’s protocol [35,36,37], instructing participants to jump as high as possible. In SJ, the participant executed a vertical jump, starting with bent legs and hands on the hips, and landing in an upright position. In CMJ, the participant had to start in an upright position, flexing the lower limbs until the thighs were perpendicular to the legs, and then quickly performing a jump up. The highest jump of two trials, with one minute of rest in between, was recorded and utilized in the subsequent analyses.

The explosive strength of upper limbs was assessed by the overhead medicine ball throw using a 3 kg ball. The participant carried the ball back behind his head and then threw it as far forward as possible. The participant could not advance beyond the line drawn on the floor before or after the ball was thrown. We measured the throw distance by a tape fastened on the floor from the front of the throwing-off line to the place of the ball drop. The longest distance reached in two successive throws was considered in the statistical analysis.

### 2.3. Statistical Analysis

Normality of data distribution was checked by the Shapiro–Wilk test. The log-transformed values of triceps skinfold were carried out before statistical comparisons.

The means and SD of the baseline data and the changes after a 10-week period, as well as the monthly growth velocity, were computed. Percentage frequency was computed for qualitative variables (weight status).

Differences in anthropometric characteristics and motor tests between the two repetitions were assessed by Student’s *t*-tests for paired samples.

The association between anthropometric changes (increments or decrements) after 10 weeks and motor tests outcomes were determined by Pearson correlation coefficients. 

Multiple regression analyses were performed to assess the effects of growth, weight status, and basketball experience on performance in motor tests. Multicollinearity was evaluated by a variance inflation factor (VIF): a value of VIF > 10 indicated excessive multicollinearity.

All statistical analyses were performed with Statistica software, version 11 (StatSoft, Tulsa, OK, USA).

A level of *p* < 0.05 was considered statistically significant.

## 3. Results

Table 1 shows the descriptive statistics (mean, SD, differences between two measurements, and percent change) of the anthropometric traits and the motor test performances of the sample players. 

The mean value of BMI falls into the category of normal weight. In our sample, 14% of subjects were underweight, 58% normal weight, 21% overweight, and 7% obese.

The relative change in SJ was the highest (7.37%), while the relative change in MUAC was the lowest (0.01%).

All motor tests significantly improved across the interval of time between the two measurements and, among anthropometric traits, weight, stature, sitting height, lower limb length, and FFM significantly increased, while UFA and %F significantly decreased. 

Table 2 shows the correlations between anthropometric changes and motor tests. A negative and significant correlation was found between changes in TST, UFA, AFI, %F, and FM with 20 m dash and *T*-test. Moreover, *T*-test was positively and significantly correlated with change in FFM and ball throw was correlated with changes in FFM and handgrip strength (right side).

No linear association was found between jumping performance and anthropometric changes.

We performed different linear regression models to evaluate the influence of basketball experience (years of practice), weight status, and growth (examined as changes in anthropometric traits) on performance (20 m dash, medicine ball throw, and *T*-test). As independent predictive variables, we selected those results that significantly correlated with each motor test, considering the multicollinearity. Only three out of five motor tests were analyzed as dependent variables, as CMJ and SJ were not linearly correlated with any change in the anthropometric variables.

The first model (Table 3) tested for 20 m dash were statistically significant and the variance explained by the selected explanatory variables was 54%. Years of experience and %F change were positively associated with speed, with a reduction of 0.06 s in time for every year of practice and for every changed percent of fat mass (unstandardized parameter B, not in Table 3). On the other hand, being obese is a negative predictor of speed. 

The second model tested for performance is the *T*-test (Table 3). The R^2^ was significant and explained 44% of the variance of the model. The results show that years of experience and %F change were significant explanatory variables of performance. Young athletes with more sports experience and with less changes in %F were the fastest in this motor test with a decrease of 15 s in time every year of basketball practice and a decrease of 30 s in time for every changed percent in fat mass.

The third model testing the explanatory variables of performance on the “medicine ball throw” test resulted significantly with an R^2^ explaining 53% of the variance (Table 4). The most predictive variables were basketball experience, weight status, and right HG change. Experience and HG change were positively associated with performance, with an increase of 12.4 cm on the throw for every year of basketball practice and an increase of 11.4 cm on the throw for every additional kg in strength. Moreover, overweight and obese children had a better performance in comparison to underweight and normal-weight players.

## 4. Discussion

In this study, we examined the 10-week anthropometric growth-related changes in a sample of preadolescent basketball players, analyzing the association of these changes, years of basketball experience, and weight status with performance, evaluated through specific motor tests. The associations between anthropometric characteristics, body composition, and successful competition in sports have already been demonstrated in adults [38,39,40,41].

In regards to basketball, anthropometric dimensions of players have been linked to both player and team success [18,42,43,44,45,46,47] and performance [43,48]. The anthropometric and physical characteristics of young players are fundamental, since parameters like body stature, body mass, stretched arms’ length, hand surface, handgrip strength, and speed, positively contribute to their performance [9,49,50]. Moreover, our results suggest that anthropometric changes, weight status, and sports experience also play an important role in performance, especially in speed and agility, among pre-teen players. 

In regards to growth variations, all the anthropometric traits increased during the considered period, except for fat parameters. One effect on the child’s growth was the increased stature due to the increase in sitting height (% change = 47%) and, particularly, in lower limb length (% change = 53%). 

The evaluation of body proportions during the pre-puberty phase is important also for the selection of basketball players, which generally happens after ten years of age and covers the period from 11 to 12 years and from 13 to 14 years, when puberty begins. Since some organic systems develop in the pre-puberty and puberty phase, this development does not always go harmoniously. Rapid lower limb lengthening does not occur simultaneously with the growth of muscles and this causes inadequate locomotive coordination. During this period, the basketball players become slower, with poorer coordination, and their mobility decreases, both with and without a ball. According to Krstevski et al. [51], this period must be considered in the basketball player’s training process; it should be revised and set on a scientific basis. 

The decrease of fat parameters could instead be connected to growth and training. 

The decline in %F was caused by the rapid growth of FFM and slower fat accumulation [52]. A decrease in %F from 11 to 12 years in boys was reported also in the study of Temfemo et al. [53]. With regard to the effect of training, significant changes in body composition with a decrease in %F and an increase in FFM were observed in both children who played sports and in overweight and obese children after a training intervention [54,55,56]. In particular, according to a Danish study [57], children engaged in sports club activities (especially ball games) show healthier body composition compared to children not active in sports clubs.

The mean value of BMI does not present significant variations over time. The majority of the subjects were of normal weight (58%), while 14% of them were underweight, 21% were overweight, and 7% were obese. The stability of BMI over the two and a half months examined can reflect body composition changes: even if FM decreased, it was compensated by an increase of FFM. 

All strength and motor performance tests significantly improved across the interval of time between the two measurements. This is in accordance with what was reported by Malina et al. [52] on the general improvement of all performance tasks with age during middle childhood and adolescence, even if without a uniform pattern for all tasks. 

Among the motor tests analyzed as dependent variables in the multiple regression analysis, 20 m dash, *T*-test, and medicine ball throw test results were significantly associated with some of the considered parameters (basketball experience, weight status, anthropometric changes). 

The sports experience is the common denominator of the three regression models, since years of practice were the most predictive variables taken from the entire analysis. 

The influence of experience was also observed in studies that considered players from different competitive levels: one factor that explains a major part of their physical differences was the years of training [12,58]. In addition, Krstevski et al. [51] noticed that basketball players who practiced three or more times per week had better anthropometric characteristics (higher body stature, greater arm span, lower values of the percentage of body fat, and a higher percentage of muscle mass) and generally achieved better results in motor tests.

Basketball experience and %F change were the most predictive variables for improvement in both the 20 m dash and in the *T*-test. On the other hand, being obese is a negative predictor of the 20 m dash: according to our findings, the higher the BMI, the slower the child runs, which is consistent with the results found by Nikolaidis et al. [59].

It is well known that body mass is an aggravating factor of speed in running; the heavier athlete has greater inertia due to larger amounts of fat, which requires greater force production per kilogram of lean mass to derive a change in flow velocity [60]. Jakovljevic et al. [61] found a significant negative correlation between the sum of skinfolds and the running speed of young basketball players. Fat acts as ballast weight because it reduces the relative power. Thus, the negative impact of fat in all regions of the body on the efficiency of locomotion is undeniable. Therefore, it is necessary to measure the body fat component to have a better frame of the physical condition of the young athletes and of its influence on performance parameters.

In regards to the medicine ball throw test, the explanatory variables of performance were weight status and right HG change, in addition to experience. According to Apostolisis and Zacharakis [49], the handgrip strength in adolescents, when stature has not been fully developed, might constitute as the most decisive factor of ball handling skills. In our study, overweight and obese children had a better performance compared to underweight and normal-weight players, and every additional kg in strength determined an increase of 11.4 cm on the throw. A positive association between strength and body size, increased BMI, and obesity, was reported in many studies [52,62,63,64,65,66]. According to Guimarães et al. [8], the medicine ball throw is very important in the selection of young athletes. In their study, one of the variables that best distinguished basketball players of different competitive levels was the medicine ball throw; therefore, they concluded that upper body explosive strength (linked to shooting, passing, and dribbling the ball) was one of the variables that magnified the differences among different group levels. Thus, understanding the factors that affect upper body strength is useful for coaches and trainers. 

Despite the repeated measures design of the study, some limitations must be highlighted: a limited sample size of only male pre-adolescents and no data on their match performance, a short follow-up time, and no control group. Conversely, one strength of this study is the use of anthropometric measurements and motor tests assessed in direct accordance to standardized techniques by repeated measurements. Further research on larger samples of pre-teens of both sexes is needed to support the promising outcomes of the proposed performance models. 

## 5. Conclusions 

In conclusion, typical basketball training activities are shown to have a positive impact on children’s health. It is also fundamental to monitor changes in anthropometric parameters that occur during the growth period; the body composition characteristics and changes, in addition to the basketball experience of young players, are crucial for their ability to play basketball and should be considered in the selection process. 

Therefore, the coaches involved in the specific age training procedures must take these results into consideration when assessing the fitness of young players and in planning effective training prescriptions.

## Figures and Tables

**Table 1:** Descriptive statistics (mean and SD), changes between two measurements, and monthly velocity of anthropometric traits and performance tests.

	I Measurement		II Measurement			Changes			
**Anthropometric Traits**	**mean**	**SD**	**mean**	**SD**	***p***	**mean**	**SD**	**(%)**	**Monthly Velocity**
Weight (kg)	47.62	11.77	48.56	11.90	<0.001	0.94	1.68	1.97	0.38
Stature (cm)	155.22	9.17	156.37	9.26	<0.001	1.15	0.61	0.74	0.46
Sitting height (cm)	79.71	4.66	80.25	4.63	<0.001	0.54	0.43	0.68	0.22
Lower limb length (cm)	75.51	5.03	76.12	5.15	<0.001	0.61	0.50	0.81	0.24
MUAC (cm)	24.20	4.01	24.21	3.78	0.988	0.01	1.04	0.04	0.00
TST (mm)	18.18	7.93	17.29	7.03	0.203	−0.89	2.62	−4.90	−0.36
UMA (cm^2^)	27.59	6.45	28.43	6.72	0.110	0.84	3.33	3.04	0.34
UFA (cm^2^)	20.31	11.49	19.34	10.28	0.037	−0.97	2.91	−4.78	−0.40
AFI (%)	39.88	10.80	38.43	9.51	0.095	−1.45	5.50	−3.64	−0.58
%F	24.91	8.52	24.09	7.93	0.017	−0.82	2.15	−3.29	−0.33
FM (kg)	12.59	7.14	12.39	6.80	0.316	−0.20	−1.29	−1.59	−0.08
FFM (kg)	35.03	6.34	36.17	6.63	<0.001	1.14	1.38	3.37	0.48
BMI (kg/m^2^)	19.62	3.63	19.72	3.67	0.356	0.10	0.72	0.51	0.04
Right HG (kg)	21.28	5.15	21.79	5.42	0.236	0.51	2.79	2.40	0.20
Left HG (kg)	20.51	5.39	21.24	5.88	0.068	0.73	2.57	3.56	0.29
**Performance tests**									
SJ (cm)	23.88	3.72	25.64	4.68	0.001	1.76	2.67	7.37	0.70
CMJ (cm)	25.59	3.79	26.97	4.61	0.006	1.38	3.12	5.39	0.55
20 m Dash (s)	3.35	0.27	3.22	0.27	<0.001	−0.13	0.17	−3.88	−0.05
Ball throw (cm)	364.27	74.86	377.02	75.00	0.001	12.76	21.02	3.50	5.10
*T*-Test (s)	11.95	1.17	11.65	1.11	<0.001	−0.30	0.41	−2.51	−0.12

MUAC: Mid-upper arm circumference; TST: Triceps skinfold thickness; UMA: Upper arm muscle area; UFA: Upper arm fat area; AFI: Arm fat index; %F: %Fat; FM: Fat Mass; FFM: Fat-Free Mass; BMI: Body Mass Index; HG: Handgrip strength; SJ: squat jump; CMJ: countermovement jump.

**Table 2:** Correlations between motor tests (second repetition) and anthropometric changes.

	SJ	CMJ	20 m Dash	Ball Throw			*T*-test			
Changes	r	*p*	r	*p*	r	*p*	r	*p*	r	*p*
Weight	0.109	0.502	0.239	0.137	0.096	0.554	0.185	0.253	−0.033	0.841
Stature	0.017	0.917	0.116	0.476	0.165	0.308	−0.110	0.497	0.171	0.290
Sit.height	0.085	0.602	0.015	0.926	0.159	0.328	−0.151	0.352	0.274	0.087
LL length	−0.057	0.729	0.135	0.405	0.066	0.685	−0.003	0.987	−0.033	0.839
MUAC	0.033	0.840	0.074	0.651	−0.134	0.411	−0.047	0.774	−0.138	0.396
TST	0.142	0.381	0.177	0.276	−0.430	0.006	−0.181	0.266	0.585	<0.001
UMA	−0.050	0.714	−0.043	0.791	0.185	0.254	0.133	0.414	0.274	0.087
UFA	0.148	0.361	0.188	0.245	−0.427	0.006	−0.232	0.150	−0.551	<0.001
AFI	0.102	0.531	0.132	0.418	−0.364	0.021	−0.105	0.518	−0.530	<0.001
%F	0.210	0.330	0.210	0.194	−0.448	0.004	−0.119	0.464	−0.582	<0.001
FM	0.110	0.501	0.166	0.305	−0.350	0.027	−0.151	0.351	−0.412	0.008
FFM	0.035	0.830	0.145	0.372	0.202	0.212	0.369	0.019	0.337	0.033
BMI	0.103	0.526	0.202	0.212	−0.140	0.388	0.162	0.318	−0.078	0.633
R HG	0.053	0.743	0.224	0.166	0.100	0.541	0.438	0.005	0.249	0.122
L HG	0.041	0.800	0.065	0.691	−0.155	0.338	0.226	0.161	0.054	0.739

MUAC: Mid-upper arm circumference; TST: Triceps skinfold thickness; UMA: Upper arm muscle area; UFA: Upper arm fat area; AFI: Arm fat index; %F: %Fat; FM: Fat Mass; FFM: Fat-Free Mass; BMI: Body Mass Index; HG: Handgrip strength; SJ: squat jump; CMJ: countermovement jump.

**Table 3:** Predictors of 20 m dash test and *T*-test: results of multivariate regression analysis.

	20 m Dash (s)				*T*-test (s)			
Predictor Variables	β	t	*p*	VIF	β	t	*p*	VIF
Years of practice	−0.389	−3.453	0.002	1.054	−0.248	−1.991	0.046	1.054
%F change	−0.485	−4.304	<0.001	1.056	−0.613	−4.913	<0.001	1.056
Weight status								
- Underweight	−0.281	−2.390	0.023	0.132	−0.216	−1.654	0.108	1.153
- Normal weight	−0.317	−2.789	0.009	0.068	−0.157	−1.244	0.222	1.072
- Overweight	−0.069	−0.600	0.553	0.101	0.065	0.509	0.614	1.112
- Obese (reference)								
R^2^	0.6032				0.5129			
Adjusted R^2^	0.5431				0.4391			
*p*	<0.0001				0.0002			

β: standardized regression coefficient; VIF: variance inflation factor.

**Table 4:** Predictors of medicine ball throw: results of multivariate regression analysis.

	Medicine Ball Throw (cm)			
Predictor Variables	β	t	*p*	VIF
Years of practice	0.311	2.694	0.011	1.082
FFM change	0.121	1.018	0.316	1.140
Right HG change	0.424	3.679	0.014	1.070
Weight status				
- Underweight	−0.307	−2.598	0.014	1.165
- Normal weight	−0.346	−2.991	0.005	1.103
- Overweight	0.185	1.568	0.127	1.144
- Obese (reference)				
R^2^	0.6029			
Adjusted R^2^	0.5284			
*p*	<0.0001			

β: standardized regression coefficient; FFM: Fat-Free Mass; HG: Handgrip strength. VIF: variance inflation factor.
